# Developing Genetic Engineering Techniques for Control of Seed Size and Yield

**DOI:** 10.3390/ijms232113256

**Published:** 2022-10-31

**Authors:** Intikhab Alam, Khadija Batool, Yuanyuan Huang, Junjie Liu, Liangfa Ge

**Affiliations:** 1 Department of Grassland Science, College of Forestry and Landscape Architecture, South China Agricultural University, Guangzhou 510642, China; 2 College of Life Sciences, South China Agricultural University, Guangzhou 510642, China; 3 Guangdong Subcenter of the National Center for Soybean Improvement, College of Agriculture, South China Agricultural University, Guangzhou 510642, China; 4 State Key Laboratory of Ecological Pest Control for Fujian and Taiwan Crops, College of Life Sciences, Fujian Agriculture and Forestry University, Fuzhou 350002, China

**Keywords:** seed-specific transcription factors, signaling pathways, seed size, seed development

## Abstract

Many signaling pathways regulate seed size through the development of endosperm and maternal tissues, which ultimately results in a range of variations in seed size or weight. Seed size can be determined through the development of zygotic tissues (endosperm and embryo) and maternal ovules. In addition, in some species such as rice, seed size is largely determined by husk growth. Transcription regulator factors are responsible for enhancing cell growth in the maternal ovule, resulting in seed growth. Phytohormones induce significant effects on entire features of growth and development of plants and also regulate seed size. Moreover, the vegetative parts are the major source of nutrients, including the majority of carbon and nitrogen-containing molecules for the reproductive part to control seed size. There is a need to increase the size of seeds without affecting the number of seeds in plants through conventional breeding programs to improve grain yield. In the past decades, many important genetic factors affecting seed size and yield have been identified and studied. These important factors constitute dynamic regulatory networks governing the seed size in response to environmental stimuli. In this review, we summarized recent advances regarding the molecular factors regulating seed size in Arabidopsis and other crops, followed by discussions on strategies to comprehend crops’ genetic and molecular aspects in balancing seed size and yield.

## 1. Introduction

The seed-producing crops are essential sources of foodstuffs, fodder, and fuel worldwide. The number and size of seeds are very imperative with respect to the evolution of various plant species for their preservation [1]. In addition, the seeds could be used to produce biofuels, which are becoming more popular as an alternative to fossil fuels [2,3]. Seeds fulfill almost 70% of the world’s food demand. Environmental factors significantly affect plant development, consequently reducing seed production. Higher yields are possible with the entire primary and secondary branches, as well as the size and pod number on every plant, flowering time, seeds number per pod, seed weight, and sometimes plant height [4,5]. The seed development in angiosperm plants begins by merging only a single sperm with a single egg cell or beside two polar nuclei, resulting in a diploid embryo or a triploid endosperm, respectively [6]. An endosperm’s single layer envelops the embryo in the mature seed of the Arabidopsis, and the seed coat encloses the endosperm, which is designed by many layers of particular maternal tissues that provide safety, enhance latency, and sprouting [7]. The development of zygotic tissues including embryo and endosperm as well as maternal components (e.g., eggs) determines seed size. Thus, the development of the embryo, endosperm, and seed envelope are the major constituents in crops, determining the seed size and weight [8]. The seed development process is divided into two phases: the morphogenesis phase, which includes cell division, endosperm and embryo development, and cotyledon differentiation; and the maturing phase, which includes embryo development at the expense of endosperm, seed dehydration, and source collection [9]. Arabidopsis seed development programs are similar to those of dicotyledon types of seed crops, i.e., canola and soybean. Moreover, early phases of seed development are identical in monocots and dicots but vary in later stages [10]. To inhabit various types of habitations, seed plants produce an enormous range of growth farms and many deviations in seed size, accompanied by seed travel ways. Moreover, after a long evolution time, plants exhibit most of the seed sizes, starting from the dust-like seeds of orchids to the 20 kg of the double coconut [11]. Seed size in flowering plants is very important from an evolutionary point of view, as well as essential for crops [12], but the molecular mechanism of seed size is not clear. Seed size is the main component in determining plant fitness and seed yield [11,12,13]. In plants, variation in seed size is an interesting phenomenon in developmental biology. Seed size is controlled by the inherent inner material regarding maternal tissues and zygotic materials [11], while the progress of the seed is affected by environmental and climatic aspects. Given the significance of seeds as the primary food source, many efforts have been made to understand seed development, size regulation, and total yield in crops. Many crop genomes have been sequenced, and genetic factors have been identified to dissect the network controlling seed growth and development [14,15].

Genetic screening is a powerful strategy widely used to identify the regulators of seed size. The advances in genome-wide association studies (GWAS) and RNA sequencing technology have been extensively applied to investigate the fundamentals of the seed size variation [16]. Plants have the good factor of huge genetic variations occurring naturally, as well as being extensively studied in many species [17]. Seed size is among the most complex characteristics, elaborately controlled by multiple positive and negative factors, constituting a well-balanced network to regulate seed size by modulating biological processes. Genes with a higher population and more density markers and several quantitative trait loci (QTLs) with a novel QTL for grain length, qGL11, were recognized in seven panicle and grain-related traits [18]. In the present review, we summarized recent advances in dissecting the regulatory network that controls seed size and yields to an extent, including the genetic factors and signaling pathways that regulate seed size in Arabidopsis and other various crops.

## 2. Genetic Factors Controlling Seed Size

Controlling seed size and weight is determined via the genetic composition of zygotic and maternal tissues. In recent times, many scientific studies have been reported to identify genetic factors with plant responses to control seed size and weight (Table 1).

In Arabidopsis, early endosperm cellularization induces diploids to pollinate tetraploids, ultimately producing smaller seeds. By contrast, late or failed endosperm cellularization causes highly abortive seeds [68,69]. The endosperm cellularization time also regulates seed size, though embryo development covers the seed cavity afterward by replacing the endosperm space. The mutant genes of the IKU-pathway, including haiku (iku1), iku2, and miniseed3 (mini3), developed smaller seeds due to precocious cellularization of the endosperm [19,20,70,71], while the SHORT HYPOCOTYL UNDER BLUEI (SHB1) gain-of-function mutants postponed endosperm cellularization, ultimately producing larger seeds [19,22,23]. Transcriptional co-activators, such as SHB1 linked with IKU2 and MINI3 promoters and stimulate their expression in Arabidopsis [22,23]. Furthermore, adjacent tissues influence endosperm development; for instance, a mutation of maternal sporophytic in TRANSPARENT TESTA GLABRA2 (TTG2) limits integument enlargement and the origins of advanced endosperm cellularization [24]. The IKU pathway interacts with TTG2 genetically by way of double mutants ttg2 and iku2, showing stronger seed phenotypes than single mutants [24]. Furthermore, a DNA topoisomerase, TOPOISOMERASE Iα (TOP1α), and an ATP-dependent RNA helicase, UP-FRAMESHIFT SUPPRESSOR 1 (UPF1), biparentally regulate the seed size (regulating TTG2). Loss of function of UPF1 or TOP1α or induces the ectopic appearance of TTG2 in antipodal cells. Genetic analysis has consistently shown TOP1α and UPF1 function upstream of TTG2. TTG2 is directly suppressed by TOP1 and UPF1 by causing a chromatin disruption [25]. Further, it has been suggested that comparative TTG2 quantity in antipodal cells to gametes regulates seed size, indicating the role of the maternal and paternal dose-dependent molecular framework. The late embryogenesis abundant (LEA) protein family named LuLEA is expressed at later stages of seed development [26]. LuLEA1 negatively regulates seed size and yield as its higher expression reduces seed size and fatty acid content in Arabidopsis. Recently, it was reported that Arabidopsis TERMINAL FLOWER1 (TFL1) acts as a mobile controller produced in the chalazal endosperm and promotes endosperm cellularization on time when moving to the syncytial peripheral endosperm, and enhances seed size by stabilizing ABA insensitive 5 (ABI5) genes [21]. Most recently, in rice, mutations in the EMBRYONIC FLOWER2a (OsEMF2a) gene resulted in delayed cellularization and autonomic endosperm development [48].

Another research group hypothesized that the seed size and length of the hypocotyl might be regulated by AtSOB3, which belongs to the SUPPRESSOR OF PHYTOCHROME B. They tested this hypothesis and demonstrated that AtSOB3-D produced lighter seeds with a shorter hypocotyl compared to WT, while a dominant-negative mutation, *AtSOB3-6-OX*, produced heavy and larger seeds with a longer hypocotyl in Arabidopsis [43]. Researchers have reported that the impact of accumulated soluble sugar on the seed size is controlled by the ANGUSTIFOLIA3-YODA (AN3-YDA) gene cascade as well as the addition of environmental and/or metabolic factors by sugar and ethylene metabolism to regulate seed mass in Arabidopsis by interacting with ETHYLENE INSENSITIVE 3 (EIN3) [28] and AN3 [27]. The ENHANCER3 OF DA1 (EOD3) exists in the sporophytic tissues of the mother plant, encodes many cytochrome P450/CYP78A6, and stimulates seed growth in plants. Overexpression of EOD3 has been shown to drastically increase the size of the seed, whereas loss-of eod3-ko function mutants produce smaller seeds. Mutants of the *BnaEOD3* gene were competently produced with stable changes by the stable transformation of the CRISPR/Cas9. These mutations were steadily transferred into the T1 and T2 generations, resulting in an accumulation of homozygous mutants with a combined loss of function alleles. The T1 line contains smaller siliques and seeds, but the higher number of seeds per silique suggests that BnaEOD3 negatively regulates seed growth and development in rapeseed [64].

Rice is one of the most essential crops, feeding a significant amount of the world’s population. Many years of studies have focused on identifying QTLs that control the grain size of rice [72]. For example, GLW7 is a major QTL that has been identified as controlling grain size and yield [49]. However, the investigation of the genetic variability of Arabidopsis has significant potential to provide insight into commercially essential traits in crops. Ren et al. (2019) identified new factors responsible for controlling seed size; CYCB1;4 is a cell cycle-related gene that encodes a cyclin protein. Growing regions are enriched in CYCB1;4 and may control the cell cycle that is positive in maternal and zygotic parts, thereby increasing seed and other organ sizes, implying that CYCB1;4 is an essential component in Arabidopsis for regulating seed size. They reported comprehensive information on GWAS in Arabidopsis related to seed size, with 38 important linked loci, and one locus interlinked with CYCB1;4. A higher level of CYCB1;4 was noted in transgenic plants, which increased seed size and grain yield in response to faster cell cycle progression, while the CYCB1;4 mutant produced smaller seeds. In short, CYCB1;4 may potentially target yield improvement in plants as the temporal and spatial expression pattern may inform functioning in both maternal and paternal tissues that are involved in absolutely organizing seed size [30]. Another study reported that overexpression of OsGW2, a QTL on chromosome 2, changes grain size and reduces Indica rice yield. The downregulation of *OsGW2* using RNAi technology resulted in larger and plump kernels by regulating cell expansion and cell proliferation in the spikelet hull [50].

Genomic imprinting regulation is very complicated and may include non-coding RNAs, DNA methylation, and histone adaptations. Many scientific studies have evaluated that the endosperm plays an essential role in divergent plant tissues, with imprinted gene expression and a unique DNA methylation [73]. The increased time for the development of endosperm between different seed parts was considered to be under the epigenetic regulator. Nevertheless, it was shown that the maternally expressed in embryo 1 (mee1) gene is imprinted in the embryo and endosperm of the maize plant [74]. Genome-wide methods relating to RNA-seq of mutual crosses in Arabidopsis [75], rice [76], and maize [77], have indicated the occurrence of numerous tentative imprints [74], and the genetic makeup of embryos. Parental imprinting of genes is caused by distinctive DNA methylations at specific loci produced by DEMETER demethylase (DME) in the maternal gametes [78], DNA Methyltransferase 1 (MET1) in the paternal gametophyte, and POLYCOMB REPRESSIVE COMPLEX 2 (PRC2)-facilitated suppression of transcription [29]. Though DNA methylation is not primarily responsible for embryo-specific imprinting, allele-definite siRNAs may induce parent-of-origin and precise genetic expression occupied by the embryo. Hypo-methylated regions in the endosperm generate maternal-specific siRNAs, which monitor ROS1-mediated DNA de-methylation of the particular area after moving into the embryo. Sequence-specific siRNAs distinguishing maternal alleles individually could result in allele specificity [79].

*CURLY LEAF* (*CLF*), which encodes a histone methyltransferase of PRC2 coordinated in tri-methylation of histone H3 Lys 27 (H3K27me3), controls a group of genes that interact with each other during embryo development [80]. Several PcG proteins such as FERTILIZATION INDEPENDENT SEED 2 (FIS2), FIS3, FERTILIZATION INDEPENDENT ENDOSPERM (FIE), FIS1/MEDEA (MEA), and MULTI COPY SUPPRESSOR OF IRA1 (MSI1) control the development of the endosperm [31]. By way of methylation of chromatin histones, repressive complexes are formed by these PcG proteins that inhibit the expression of involved genes controlling developmental processes. Numerous PcG constituents can be imprinted, and the functions of FIS complexes in plants are manifested after pollination. Moreover, *fis* mutants decrease endosperm cellularization and, ultimately, the size of seeds. The function of FIS is mediated by AGL62, which belongs to the agamous-like MADS-box proteins, whereas in *fis* mutants, *AGL62* expression is suppressed, leading to endosperm cellularization failure [32]. Regarding the FIS gene family, FLOWERING WAGENINGEN (FWA), *MEA*, and *MEA* genes are maternally expressed; however, *MADS-box I like PHERES1* (*PHE1*) and a FIS PcG complex are presumed to be paternally expressed. Seed growth is terminated due to peak propagation of the endosperm without embryo development in *medea* (*mea*) mutants [81]. The FIS proteins, including MEA, FIE, and FIS2, strongly regulate the MADS-box gene *PHE1* expression. *PHE1* expression was normal after fertilization in wild-type plants (WT), whereas *fis* mutants showed higher expression until seed abortion, indicating that FIS-class proteins deregulate *PHE1* expression after fertilization to prevent seed abortion [33]. Recently, using CRISPR/Cas9-based genome editing, it was found that the MADS-box TF genes MADS78 and MADS79 are very important for regulating endosperm cellularization and early seed formation in rice [55]. A single MADS78 or MADS79 knockout mutant displayed early endosperm cellularization, while double mutants hindered seed development and produced no viable seeds [55].

DNA methylation plays a significant role in seed development as it may help increase seed size and weight [82]. It also plays an important part in plant genome stability and developmental processes [83]. Chinese super hybrid rice LYP9 (Liangyou Peijiu) is a successful hybrid rice variety with high heterosis [84]. This indicates that DNA methylation in hybrid rice seeds plays a key function in the initiation and maintenance of heterosis [85]. Xiao et al. (2006) showed that methylation of *FIS2* and *MEA* is facilitated by *MET1* containing DNA methylase, methylating CpG islands, and a genomic cytosine base. A *met1* mutation in the maternal genome increases seed size due to maternal hypomethylation and delays endosperm cellularization [37]. By contrast, *met1* mutations in the paternal genome lead to early cellularization of the endosperm and decreased seed size, thus confirming exclusive maternal control of *MEA* expression, which provides insights into the evolution of imprinting in plants. Similar findings were also obtained in reciprocal crosses with parents with mutations in *ddm1. FIS2* [38], *FWA* [39], and *MPC* [40], which are imprinted genes that regulate parental imprinting by DNA methylation [86]. However, the effects of paternal imprinting are minor or non-existent. Another study in Arabidopsis revealed that a deviation in the paternal alleles regulates the growth of seeds, and these deviations occur in seeds when mutants with the maternal *mea* allele showed loss of function of this gene, indicating that paternal MEA may compensate maternal MEA deficiency during seed development [41]. During the developmental phase of rice seeds, genome-wide DNA methylome analyses showed higher frequencies of DNA methylation in embryos than in the endosperm, and non-transposable element regions were more variable than transposable ones during endosperm and embryo development [87]. Many epigenetic regulatory mechanisms participate in seed development; however, the regulatory mechanisms underlying major genome imprinting processes are not yet clear. Thus, integrated exploration of genome-related DNA methylome differences, histone amendment, transcriptomics, and cleavage positions of miRNA in developing seeds through current genomic approaches, including degradome and bisulfite sequencing, is required for a better understanding of epigenetic mechanisms underlying seed development. The turgor pressure of the endosperm drives seed expansion during the early seed development [88], whereas later, turgor pressure decreases during endosperm cellularization, which is controlled by POLYCOMB REPRESSIVE COMPLEX 2 (*PRC2*) [42].

MicroRNAs (miRNAs) have become an important part of the basic molecular network of important agronomic seed traits in various plant species. The loss of the specific gene functions that control miRNA biogenesis results in defects in seed development [89]. MicroRNA160 targeting auxin response factors in cotton and suppression of *miR160* resulted in a smaller seed size in cotton [62]. Another study examined the potential role of miR156a in linseed flax (*Linum usitatissimum* L.) by decreasing seed oil content in transgenic lines compared to WT plants [90]. In addition, miRNAs also control embryogenesis by regulating transcription in maize [56]. Recently, 72 existing miRNAs and 39 recently identified miRNAs were discovered to be expressed in the growing seeds of legumes (*Phaseolus vulgaris* L.), and their involvement was more prominent throughout late embryogenesis and desiccation. Numerous TF families have been identified as known miRNA targets, including ARF, HD-ZIP, NF-Y, and SPL, and a majority of new miRNA targets were anticipated to be expressed as functional proteins [91]. In addition, small interfering RNA (siRNAs) can also induce transcriptional gene silencing, which may be associated with epigenetic control of seed expansion. Plant-specific RNA polymerase named RNA polymerase IV (Pol IV) is involved in the production of siRNAs [92]. Genome-related monotonous elements are used to produce p4-siRNAs, and some are interlinked with exclusive regions, comprising numerous characterized imprinted loci that may have a significant function in the initiation or continuation of imprinted gene expression [93]. Pol IV-base p4-siRNAs are produced from transposon elements present in the genome and are expressed exclusively in the endosperm tissues [44].

Quantitative trait locus analysis is useful for providing information on seed size control and other traits which may be relevant in plant breeding. Recently, 39 QTLs were identified on different chromosomes in common beans, and one major QTL, SL9.1 ^GA^, was found to control seed length [63]. Three epistatic QTLs (qSL-13-3_zy_, qSL-13-4_zy_, and qSW-13-4_zy_) were found to regulate soybean seed size and yield [65], and many well-categorized QTLs have been recognized in different plants, including *GS3, qSW5*, and *qGL7* in rice [51,53], *GSI*, *qZmGW2-CHR4*, *zMgs3*, and *qZmGW2-CHR5* in maize [57,58,59] and *TaGW2* in wheat [61].

Moreover, many QTLs involved in seed size control have been detected in other crops, although their functions have not been categorized [94,95,96]. Genes with higher abundance and density markers and 43 QTLs were recognized to be linked with seven panicle- and grain-related characteristics. For example, the new locus qGL11 enhances rice grain weight and length, and it occurs in a chromosome segment substitution line (CSSL) obtained through segregation of the population; moreover, it mapped exceptionally well onto a 25-kb sequence that comprises the IAA-amido-synthetase gene *OsGH3.13* [18]. A recent study investigated QTL regions at the 168.37 kb chromosome fragment with regard to the SNPs Aradu_A07_1148327 and Aradu_07_1316694. Twenty-two genes were predicted in this region, with two genes of interest, Aradu.RLZ61 and Aradu.DN3DB, encoding a transcriptional regulator, an F-box SNEEZY (SNE), and STERILE APETALA-like (SAP), respectively. These genes play a critical role in regulating seed and fruit size [67]. Moreover, *MINI SEED 2* (*MIS2*) regulates grain size by coordinately controlling epidermal cell number and cell size, and microscopic analysis showed that *mis2* mutant rice revealed reduced cell size of spikelet epidermis but higher cell numbers [54]. The *MIS2* encodes CRINKLY4 (CR4), the receptor-like kinase through Map-based cloning, which exhibited the strongest expression during panicle development [97].

In rice, the *LARGE1* gene encodes the MEI2-LIKE PROTEIN4 (*OML4*) and negatively regulates seed size through terminating cell enlargement in spikelet hulls. Over-expression of *OML4* results in smaller and lighter seeds, whereas loss of function of *OML4* leads to larger and heavier grains [52]. In eukaryotes, cyclin-dependent kinases (CDKs) and their governing subunit cyclin (CYC) control the cell cycle progression [34]. CDK inhibitors (CDKIs) are inhibitors of CDK/CYC complex action and were thus the principal cell cycle controllers. In plants, two types of CKIs have been discovered and categorized: SIAMESE/SIAMESE-RELATED (SIM)-related suppressors and INHIBITOR OF CDC2 KINASE/KIP-RELATED (ICK/KPR) [98]. Central-cell *rbr* mutants have superfluous nuclei, indicating failed cell cycle arrest [99]. Moreover, irregularities in *rbr1* maternal gametes decrease cell propagation in the integument and stop further development of the seed envelope [47]. Furthermore, a complex of MUTICOPY SUPPRESSOR OF IRAI (MSII) RBR1, which stops the expression of *MET1,* is responsible for activating imprinted genes present in germ cells throughout megagametophyte development [35]. Seed termination was observed in the tri-mutant *cycd3-1:2:3* (CYCD3 belongs to the CYCD gene family), because of late cell propagation during embryo development. However, cell division stimulated by the trans-activation of *CYCD3:1* or *CYCD7:1* in the endosperm and embryo results in fatal embryo deficiencies [100]. Directed upregulation of *CYCD7:1* in the endosperm and in the central cell, which resulted in escape from the cell cycle in the central cell, and enhanced endosperm propagation at the syncytial stage, resulting in a larger seed. Nevertheless, death occurred in a few larger seeds, indicating an incomplete seed termination [46]. The *ENO* gene is strongly expressed in plants encoding glycolytic metalloenzyme enolase (ENO2) that boosts the dehydration of 2-phospho-D-glycerate (2-PGA) to phosphoenolpyruvate (PEP) [101]. Three *ENO* genes have been identified in Arabidopsis, including *At1g74030* (*ENO1*), *At2g36530* (*ENO2*), and *At2g29560* (*ENO3*) [102]. Sub-cellular occurrence of isoforms of enolase showed that ENO1 and ENO3 are situated in the chloroplast and cytoplasm, while ENO2 has been detected in the nucleus and cytosol [103]. In addition, mutations in AtENO2 decreased the size and mass of seeds with a reduced concentration of cytokinin. Carbohydrate data analyses and RNA sequencing showed that metabolism pathways, particularly regarding the secondary metabolism, occurred in *AtENO2 T-DNA* mutants [36]. Furthermore, AtENO2 cooperated with Arabidopsis basic-leucine zipper 75 (AtbZIP75) as a substitute for AtMBP-1 [36]. Therefore, AtbZIP75 may contribute to seed development. This study clarified the novel function of AtENO2 in seed growth and improvement and proposed a good target gene for gene manipulation for the purpose of plant breeding. Recently, two SWEET homologs, i.e., *GmSWEET10a* and *GmSWEET10b*, were found to simultaneously affect seed size, oil quantity, and protein content in soybean, suggesting that seed size and oil constituents can be controlled by adjusting the combination of both alleles [15]. A different study demonstrated that the reproductive functions of this plant, including fertility, seed size, and yield, can be controlled by C-TERMINALLY ENCODED PEPTIDE RECEPTOR1 (CEPR1) [45]. The authors showed that two *cepr1* knockdown mutants produced smaller seeds and reduced yield (by 88–98%). In Arabidopsis, *BZR_1_* controls organ size via the BR signaling pathway. Recently, studies have shown that overexpression of *ZmBZR_1_* in Arabidopsis causes increased organ and seed size. Moreover, *ZmBZR_1_* is attached to the promoter of *GRACE* and *KRP6* to control their expression and regulate seed size [60].

## 3. The Role of Signaling Pathways in Seed Size Regulation

### 3.1. The Ubiquitin-26s Proteasome Pathway

**Figure 1:**
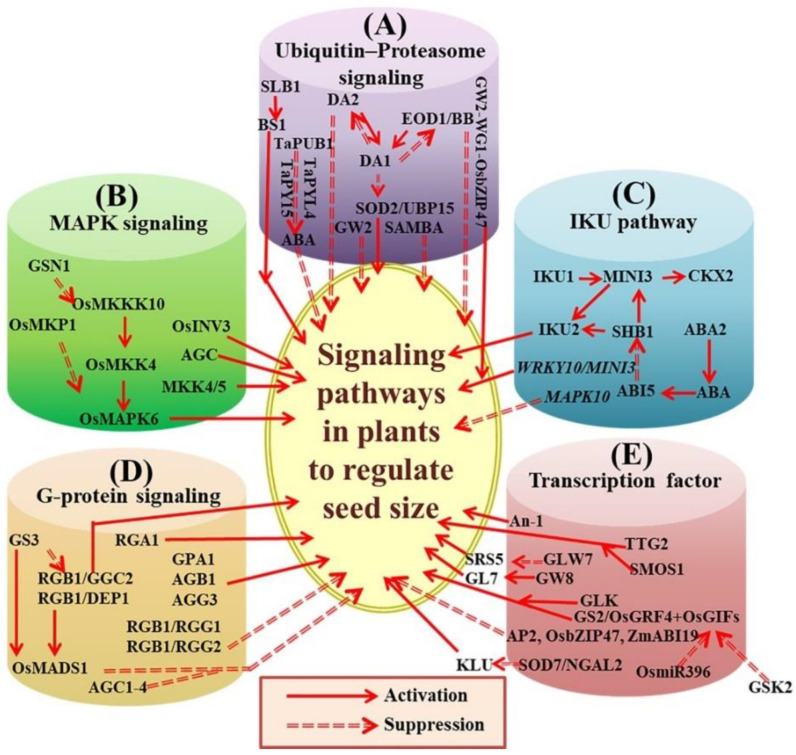
Signaling pathways and their correlated molecules involved in seed size regulation. (**A**) Ubiquitin–26s proteasome pathway (**B**) MAPK signaling pathway (**C**) IKU pathway (**D**) G-protein signaling pathway (**E**) Transcription regulatory factors.

The ubiquitin–26S pathway regulates the size and development of seeds; the genes regulating the ubiquitin–proteasome pathway are shown in Figure 1A. Ubiquitination and/or protein degradation processes by the proteasome 26S pathway are an important post-translational protein turnover mechanism in plants [104,105,106]. The ubiquitin–proteasome system, which is highly controlled, is involved in the regulation of all aspects of plant life and governs numerous developmental and stress-related activities mediated by hormone signals [107]. Ubiquitin (Ub) is a protein of 76 amino acids that affect the target cellular proteins via a multistep reaction including three enzymes, E1 enzyme (Ub-activating), E2 enzyme (Ub-conjugating), and E3 enzyme (Ub ligases), in the ubiquitin–proteasome system (UPS) [108]. Ubiquitination specificity is largely regulated by E3 ligases, which recognize specific substrates and catalyze the bond between the Ub and substrates [108]. E3 ligases are important regulatory components in the ubiquitin-dependent pathway because they mediate substrate specificity [109]. The ubiquitination pathway participates in seed size regulation; ubiquitin receptor, E3 Ubiquitin ligase, ubiquitin-specific protease, 26S proteasome, plant-specific APC/C regulatory factors, and ubiquitination pathway interaction proteins, etc., all play a central function in regulating seed size and development [13,110,111]. In Arabidopsis, ubiquitin receptors such as DA1 and DA1-related protein (DAR1) regulate the size of seeds by restraining cell propagation in the maternal integument [112]. The DA2, E3 Ub-ligases, and BIG BROTHER (BB)/ENHANCER OF DA1 (*EOD1*) function together with DA1 to limit seed size and the development of other plant parts [110,113], indicating that E3 ubiquitin ligases and DA1 may function in the same complex or have mutual downstream targets. Supporting this concept, DA1 strongly co-operates with DA2 in vitro as well as in vivo [110]. E3 ubiquitin ligase shows specific substrate relationships between DA1 and DA2, and it may help to identify the precise substrates of DA1 with regard to degradation. Genetic studies have revealed that BB/EOD1 and DA2 function in diverse ways [110], indicating that variant substrates may be targeted by them for degradation. In rice, grain width and weight 2 (*GW2*) shows sequence resemblance with DA2, encoded by a QTL regulating seed size [13]. GW2 negatively affects the size of grains by controlling cell propagation in spikelet casings. Homologs of GW2 are also associated with seed size regulation in maize and wheat crops [58,61], suggesting that *GW2* retains preserved functions of seed size regulation. In Arabidopsis, *SUPPRESSOR OF DA1* (*SOD2*) encoding UBIQUITIN-SPECIFIC PROTEASE 15 (UBP15) is maternally responsible for enhancing seed development. Plants with *sod2/ubp15* mutations show reduced seed size and other organs because of the reduction in cell propagation and epistatic effects on DA-1 [114,115]. In Arabidopsis, DA1 interacts physically with UBP15 and modifies its durability, suggesting that UBP15 is the downstream target of DA1. Further study showed that UBP15 individually affects EOD1 and DA2 to regulate grain size, specifying that other unidentified E3 ubiquitin ligases may ubiquitinate UBP15. Moreover, in Arabidopsis, the anaphase-promoting complex/cyclosome (APC/C) ubiquitin ligase and the 26S proteasome, along with many other factors such as RPT2 and SAMBA, affect seed development [116,117,118]. SAMBA mutations, a negative regulator of APC/C, result in a larger seed size [117]. The mutation in SAMBA markedly increases seed size and structure in eod1-2 da1-1 phenotypes [119], implying that SAMBA may exert substantial effects or act as a complex identical to EOD1 and DA1 regarding grain size regulation. The important QTL qSW5/GW5 is responsible for seed thickness; it is located on chromosome 5 and controls the size of grains by suppressing cell propagation in spikelet casings in rice [120]. GW5 reacts to ubiquitin in vitro, signifying that it may act as a ubiquitin–proteasome pathway [121]. In rice, GW5 may function separately from GW2 to regulate grain width [122]. Histone ubiquitylation controls the transcription of *DA1/DA2*, which affect seed size. OTU1 de-ubiquitinase de-ubiquitylates DA1/DA2 chromatin and acts as an epigenetic transcriptional suppressor of the *DA1/DA2* genes. OTU1 is nucleocytoplasmic and plays a role in nuclear and cytoplasmic functions [123]. *SMALL LEAF AND BUSHY1* (*SLB1*) encodes the F-box protein, a fragment of the SKP1/Cillin/F-box E3 Ub-ligase complex. *SLB1* regulates the growth of plant organs and secondary branches by regulating the stability of BIG SEEDS1 (BS1), leading to increased leaf and seed size in soybean and *Medicago truncatula* [124]. A different study reported that the regulatory complex GW2–WG1–OsbZIP47 regulates grain size in rice [27]. GW2, WG1, and OsbZIP47 work together to control grain width and length through the GW2–WG1–OsbZIP47 regulatory module. Specifically, *WG1* encodes a glutaredoxin protein and promotes cell proliferation, leading to enhanced grain growth. WG1 acts as a co-repressor with ASP1 and interacts with the transcription factor (TF) OsbZIP47 to terminate its transcription. OsbZIP47 suppresses seed growth by reducing cell proliferation [28]. Moreover, the E3 Ub-ligase *TaPUB1* reduces the seedlings’ sensitivity to the abscisic acid (ABA) by interacting with TaPYL4 and TaPY15 (involved in ABA signal transduction and inducing their degradation), resulting in smaller grain size and yield; this suggests that TaPUB1 is a negative regulator of seed development [125].

### 3.2. The Mitogen-Activated Protein Kinase (MAPK) Signaling Pathway in Plants

MAPK is responsible for many transduction pathways, including hormone signaling and stress responses [126,127,128]. The genes regulating the MAPK signaling pathway are shown in Figure 1B. A MAPK cascade comprises three kinases: MAPK, MAPK kinase (MAPKK), and MAPKK kinase (MAPKKK). MAPKKKs activate and phosphorylate MAPKKs in response to an external stimulus signal, and then the activated MAPKs phosphorylate a variable number of downstream target substrates, including transcription factors, chromatin remodeling factors, kinases, or different enzymes, resulting in transcriptome and proteome reprogramming in the entire cell. The successive phosphorylation of MAPK proteins and their substrates is critical for MAPK cascade-mediated interactions and signal transduction [129]. Several components of the MITOGEN-ACTIVATED PROTEIN KINASE (MAPK) cascade have previously been identified as key regulators of seed development [130]. The *small grain 1* (*smg1*) encoding MAPKK4 mutants generate short grains in rice because of reduced cell proliferation in spikelet exteriors [131]. In addition, a previous study suggested that alterations in OsMAPK6 result in short grains, which resemble those produced by *smg1* mutants [132]. Furthermore, loss of function mutations in OsMKP1 produce larger grains, while OsMKP1 overexpression generates smaller grains. OsMKP1 regulates rice grain size by limiting cell proliferation in grain hulls. OsMKP1 directly interacts with and deactivates OsMAPK6 [133]. Moreover, OsMKK4 is physically co-related with OsMAPK6 [132]. Therefore, the OsMKK4–OsMAPK6-controlling module plays an essential role in controlling seed size in rice. It is difficult to investigate the upstream OsMAPKKKs of OsMKK4 and downstream substrates of OsMAPK6 regarding the control of grain size. Furthermore, OsMKK4 and OsMAPK6 affect brassinosteroid (BR) reactions and many BR-related genes’ expression [131,132], suggesting a link between the MAPK pathways and BRs with respect to seed development. Guo et al., 2018 demonstrated that GSN1 functions as a negative regulator of the OsMKK10–OsMKK4–OsMPK60 cascade by inducing precise dephosphorylation of OsMPK6 to coordinate the trade-off between grain size and grain number per panicle [134].

The leucine-rich repeat (LRP)-receptor kinases ER, ERL1, and ERL2 stimulate fruit growth through signaling pathways regulated by EPFL9 in the carpel wall. EPFL2 expression controls the spacing of ovules in the wall of the carpel, and in inter-ovule spaces, this is controlled by ERL1 and ERL2, which may facilitate equal distribution of resources [135]. This may provide an understanding of trade-offs in rice panicle expansion and constitute a basis for increasing crop yield. The novel AGC protein kinase AGC1-4, belonging to the subfamily of AGC VIIIa, encodes a serine–threonine kinase and regulates seed size. Seeds with higher expression of AGC1-4 are smaller, whereas *agc1-4* mutant seeds were significantly larger [136]. A recent study reported that *OsINV3* is a positive seed size regulator in rice [137]. They showed that overexpression of *OsINV3* increased grain size, while loss of its function reduced grain size, in comparison with WT plants. *OsINV2* is a homolog of *OsINV3* and has no function in seed size regulation by itself; however, both *OsINV2* and *OsINV3* play roles in sucrose metabolism in sink organs and increase seed size [137]. In Arabidopsis, the function of the novel gene *ZmRLK7*, which belongs to the LRP receptor-like protein kinases (LRR-RLKs) isolated from maize, was investigated through ectopic expression to examine its effects on plant development [138]. Most recently, in Arabidopsis, the lectin receptor-like kinase LecRK-VIII.2 has been found to regulate seed production by organizing seed size, seed number, and silique. Lecrk-VIII.2 mutants produce fewer seeds but more seeds and siliques, resulting in a higher yield. On the other hand, overexpressing of LecRK-VIII.2 produces larger seeds but fewer seeds and siliques, resulting in yields comparable to wild-type plants [139]. MPK3 and MPK6 double mutants are toxic to the embryo, but single MPK6 mutants exhibit a variety of abnormal phenotypes related to seeds, similar to those seen in mutants of the kinases MAPK KINASE 4 (MKK4) and MKK5, which function upstream of MPK3 and MPK6, respectively. Given the importance of MPKs in seed development and their function in RLK signaling. (Xiao et al., 2021) investigated whether MPK3 and MPK6 could act downstream of LecRK-VIII.2 [139]. All these findings suggested that the phosphorylation of MPK6 and not MPK3 corresponds with the expression levels of LecRK-VIII.2 in the seed. According to (Xiao et al., 2021), LecRK-VIII.2 promotes growth throughout development. Furthermore, this important receptor positively promotes seed growth from maternal sporophytic tissues during seed development by activating expansions, and possibly through the MAPK cascade and MPK6. This discovery adds to the body of evidence supporting the importance of LecRKs in developmental processes and opens up new paths for advanced research, such as investigating the signal that LecRK-VIII.2 perceives. Understanding the mechanism behind the greater yield in LecRK-VIII.2 KO plants, despite an overall loss in plant growth, will be fascinating. Is it feasible that a plant with lower vegetative biomass has more available resources for seed production before it reaches senescence? Is it possible to manipulate this signaling pathway to generate crops with larger seeds, stronger plants, and higher yields?

### 3.3. The IKU Pathway

Seed size controlled through an IKU signaling pathway loss-of-function mutation in the VQ motif protein HAI-KU1 (IKU1), receptor kinase IKU2, WRKY TF MINI-SEED 3 (MINI3), and the leucine-rich repeat (LRR) decreased seed sizes due to advanced endosperm cellularization. The genes regulating the IKU pathway are shown in Figure 1C. The embryo and endosperm genome, instead of maternal genetic factors, determine the size of the seed phenotype in these mutants [20,22]. Transcriptional co-activators such as SHORT HYPOCOTYL UNDER BLUE 1 (SHB1) have been shown to link with IKU2 and MINI3 promoters and stimulate their expression in Arabidopsis [22,23]. Seed size is regulated by endosperm growth through the signaling pathways of IKU1, IKU2, SHB1, and MINI3 [140]. MINI3, which is associated with the cytokinin oxidase (CKX2) promoter stimulates the initiation of CKX2 expression and is responsible for controlling endosperm growth [110]. Higher expression of CKX2 enhances seed size in iku2 seeds, confirming the role of cytokines in down-regulating the IKU pathway with regard to seed size regulation [110]. ABSCISIC ACID-INSENSITIVE 5 (AB15) is a TF that directly attaches to the upstream region of SHB1 and inhibits its expression [141]. ABA controls the development of seeds by ABI5-facilitated transcription and is regulated by SHB1 in the endosperm; thus, the mutants abi5 and ABA-deficient 2 (aba2) abi5 produce the larger size of seeds [141]. In combination with cytokinin and ABA signaling, the IKU pathway affects endosperm growth to control seed size in Arabidopsis and other plants. In Arabidopsis, the function of the new gene *ZmRLK7*, which belongs to the LRP receptor-like protein kinases (LRR-RLKs) isolated from maize, was investigated using ectopic expression [138]. The roles of *WRKY10/MINI3* and *MAPK10* in endosperm development were examined, and they appeared to function in opposite patterns. Furthermore, *mapk10* mutants consistently produce larger seeds, and seed size is positively regulated by *WRKY10/MINI3* [142].

### 3.4. The G-Protein Signaling Pathway

In plants and animals, G-protein signaling regulates many functions related to growth and development. Molecules regulating G-protein signaling are shown in Figure 1D. Many cell surface G-protein-related receptors are connected to intracellular effectors by the G-protein complex. Ligands activate receptors, and effectors control different cellular responses [143]. G-protein complexes comprise Ga, Gb, and Gg subunits, and G-protein-coupled pathways transfer signals through membrane-bearing receptors and heterotrimeric compounds to downstream effectors. Thus, mutations in Ga (GPA1) or Gb (AGB1) result in small leaves and flowers in Arabidopsis [144,145]. Over-expression of the Arabidopsis atypical Gg (AGG3) stimulates the growth of seeds and organs by enhancing cell propagation, and loss-of-function mutations in AGG3 lead to short seeds and organs [146,147]. AGG3 overexpression in *Camelina sativa* resulted in increased seed size, confirming the function of AGG3 in the regulation of seed size [148]. Likewise, loss of function or functional suppression of rice Ga (RGA1) or Gb (RGB1) reduces rice grain size [149,150,151]. Similarly, the knockdown of the RGB1 gene in rice delayed seed development, and lowered seed weight and starch accumulation [152]. Two QTLs affecting the size of grains and panicles are Rice GRAIN SIZE 3 (GS3) and DENSE AND ERECT PANICLE 1 (DEP1), respectively [146,147]. However, long seeds are produced after the loss of function due to increased cell propagation, whereas short grains are produced in response to GS3 or DEP1 gain-of-function alleles [153,154,155]. It may be possible that AGG3 in Arabidopsis and GS3 and DEP1 in rice have various cofactors or effectors, suggesting differential effects on seed size. A minor QTL, Small grain 3 (SG3) encoding an R2R3 MYB protein is associated with a major QTL grain size 3 (GS3) and negatively controls rice grain length. The γ subunit of a G protein encoded by *GS3* competitively interacts with Gβ, leading to reduced grain length [156,157]. DEP1 also encodes a γ subunit of a G protein, and GS3 appears to act as a cofactor of OsMADS1 in the control of grain size through the regulation of common target genes [158]. Recently, a study introduced a serine–threonine protein kinase AGC1-4 belonging to the subfamily of AGC VIIIa [136]. AGC1-4 overexpression causes a reduction in seeds, whereas *agc1-4* mutants produce significantly larger seeds compared with WT plants. AGC1-4 regulates seed size by regulating the number of embryonic cells [136]. Recently, heterotrimeric G-protein mutants of rice were produced using CRISPR/Cas9 gene editing. The *gs3* and *dep1* mutants produced more favorable agronomic traits than WT plants, whereas the *rga1* mutation resulted in a dwarf phenotype, leading to an extreme reduction in grain yield. The heterotrimeric G-protein β subunit, RGB1, plays a significant role in plant development, and *rgb1* mutants show suppressed growth and development of the embryo [159]. Most recently, the E3 ligase gene Chang Li Geng 1 (CLG1) has been shown to negatively regulate grain length by targeting the Gγ protein GS3, and thus control grain size. CLG1 overexpression increased grain length, and CLG1 mutations with variations in three essential amino acids decreased grain length. CLG1 directly interacts with and ubiquitinates GS3, which is then destroyed via the endosome degradation pathway, resulting in improved grain size [160]. More studies are required to comprehensively elucidate the functions of G-protein signaling in seed size regulation.

### 3.5. Transcriptional Regulation

Particular transcription factors, i.e., TRANSPARENT TESTA GLABRAA2 (TTG2) and APETALA 2 (AP2), affect seed development in Arabidopsis [24,161,162,163,164]. Genes regulating transcription factors are shown in Figure 1E. The transcriptional repressor NGATHA-like (NGAL-2)/SUPPRESSOR-OF-DA1 (SOD7) and its homolog NGLA3/DEVELOPMENTAL-RELATED-TARGET-IN-THE-APEX 4 (DPA4) have been predicted to strongly limit Arabidopsis seed development [165], and *sod7-2 dpa4-3* mutants showed enhanced cell proliferation in the maternal integument, resulting in big seeds [165]. The Arabidopsis *AINTEGUMENTA* (*ANT*) gene is associated with integument and organ growth regulation [166,167], and it encodes APETALA2-like TF *ant* mutants that exhibit smaller leaves, reduced flowering, imperfections in integument origination, and development of ovules. However, Arabidopsis and tobacco plants with higher expression of *ANT* showed higher production of leaves and flowers because of stimulated cell proliferation, and consequently, they produced larger seeds. An-1 encodes a basic helix–loop–helix protein, which regulates cell division. Transgenic studies have confirmed that An-1 positively regulates awn elongation, but negatively regulates grain number per panicle [168]. APETALA2 is a member of the AP2/EREBP (ethylene-responsive element binding protein) group belonging to the family of TFs that plays a significant role in the specification of floral organs in Arabidopsis [169], and it is responsible for seed size regulation [161,163]. The production of seeds is higher in Arabidopsis *ap2* mutants than in WT plants, regardless of the genotype of the pollen donor, signifying that AP2 is a maternal factor controlling seed size [161]. Tissue-specific epigenetic mechanisms control gene expression and transcriptional regulators at specific stages, and epigenetic processes allow genomic imprinting; thus, the allele’s expression after pollination depends on the parent of origin. The “parental conflict theory can describe the importance of biological imprinting”. A substantial barrier prevents resource distribution from mothers to offspring, and the main function of the paternal genome is to quickly deliver maternal resources to embryos harboring the paternal genome. However, the maternal genome attempts to distribute resources equally between offspring by downregulating the effects of the paternal genome [170]. Further, *ap2-10* (+/–) seeds generated by an *ap2* mutant are bigger than WT seeds, even in flowers pollinated with WT pollen, but seeds are smaller compared to *ap2-10* (–/) seeds of mutant flowers pollinated with *ap2* pollen [163], indicating that *AP2* functions maternally and zygotically to regulate seed size. In support of this concept, seeds yielded by cross-pollination of *35S:AP2* antisense transgene mutants with WT flowers were bigger than WT seeds and smaller than *ap2* mutant seeds [163]. For instance, SMOS1 (SMALL ORGAN SIZE 1) encodes an auxin-regulated APETAL2-type transcription factor and is positively regulated by OsARF1 in rice [171]. The loss of function of SMOS1 causes pleiotropic developmental phenotypes, including small seed size [171,172], suggesting a crucial role for SMOS1 in seed size regulation.

In *transparent testa glabra*2 (*ttg2*) mutants, yellow seeds are produced due to insufficient proanthocyanidin production and deposition of mucilage in the seed coat by the mutant [24]. The *ttg2* mutants’ seeds are round and small, causing reduced cell length in the integument, in contrast to WT seeds. Reciprocal crossing trials showed the effects of *ttg2* on seed size in a strongly maternal sporophyte. The *ttg2* mutation also induced advanced endosperm cellularization, resulting in endosperm size reduction. *TTG2* encodes a WRKY family TF which regulates many steps of tannin synthesis [162], and, as several products of the tannin synthesis pathway may affect the cell wall and its capability to increase, mutations in *TTG2* may reduce the elongation ability of the cell wall [24]. The *ALM1* gene encodes Golden 2-like (GLK) a member of the GARP subfamily of Myb TFs, and *alm1.g* and *alm1.a* mutants revealed a decrease in 100-grain weight by 15.8% and 23.1%, respectively [173,174].

Several genes, including GROWTH-REGULATING FACTOR (GRF) genes, encode DNA binding transcription factors that interact with and form a functional transcriptional complex with the transcription cofactor GRF-INTERACTING FACTOR (GIF) [175]. In this functional unit, GIF operates to recruit SWI/SNF chromatin remodeling complexes to their target genes so that they can be transcriptionally activated or inhibited by GRF. GRF expression is post-transcriptionally inhibited by microRNA (miR396) [175].

It is precisely the miR396–GRF/GIF module that influences a wide range of essential plant growth and development traits that could have agricultural implications. However, the most convincing results demonstrating the agronomical values of the miR396–GRF/GIF system come from a set of many studies that independently identified a rare naturally occurring allele of OsGRF4 from *Oryza sativa* landraces with larger grain size in various genetic backgrounds [176,177,178,179,180,181]. Overexpression of OsGIF1 also improved grain size [179,180,182,183,184]. Likewise, the expression of a mimic transgene that binds and inactivates miR396 in rice, a strategy also used in Arabidopsis and other species, can increase the yield. Together with the fact that effects similar to those were artificially introduced in Arabidopsis, these findings demonstrate the transferability of knowledge from models like *A. thaliana* to crops, and the general importance of the miR396–GRF/GIF module in other plant species.

The NACs are transcription factors (TFs) that are specific to plants and are involved in many developmental processes [185]. For example, NAC-like TF EjNACL47 might be linked with the larger organ size in triploid loquat. Furthermore, ectopic expression of EjNACL47 results in larger organs i.e., leaves, flowers, and siliques in Arabidopsis, implying a positive role in organ enlargement [186]. In rice, the *ONAC020*, *ONAC026*, and *ONAC023* genes encoding NAC TFs express specifically during seed development. They have a strong relationship with seed size or weight as sequence changes in the upstream regulatory region [187]. The KIX–PPD complex regulates maternal integument growth and, consequently, regulates seed size through cell propagation and development. In Arabidopsis, the transcription factor MYC3/4 reacts with PPD1/2 and KIX8/9 to form the KIX–PPD–MYC product. The KIX–PPD–MYC product suppresses the expression of the *GIFI* promoter when interacting with the G-box region located in the *GIFI* promoter, and controls seed size [188]. Promoters of the grain filling-specific TF gene Opaque2 (O2), which regulates the factors known as B3 domain TF and ZmABI19, directly fuse with the O2 promoter for trans-activation and affect the developmental process of the endosperm and embryo, resulting in smaller seeds [189].

The KIX domain-containing protein family acts as a negative regulator of cell propagation in plants [190]. Loss of function in GmKIX8-1 mutants significantly increases the size of above-ground organs, including leaves and seeds, with increased cell propagation and *CYCLIN D3;1-10* expression. In addition, molecular analysis of soybean germplasms showed that increased expression of the qSw17-1 QTL with a large seed phenotype is caused by decreased expression of GmKIX8-1 [66]. A different study reported that a regulatory complex, GW2–WG1–OsbZIP47, regulates rice grain size. *GW2*, *WG1*, and *OsbZIP47* together regulate grain width and length through the GW2–WG1–OsbZIP47 regulatory module. Specifically, *WG1* encodes a glutaredoxin protein and promotes cell proliferation, leading to enhanced grain growth. WG1 acts as a co-repressor with ASP1 and interacts with the transcriptional factor OsbZIP47 to terminate its transcription. OsbZIP47 suppresses seed development by reducing cell proliferation [28]. During development, the shape of the grain is controlled by many genes that interact with one another. GW8 is a member of the SBP transcription factor family. When it is overexpressed, it promotes cell growth and grain filling, which leads to wider grains and higher yields. In addition, GW8 also interacts with the GL7/GW7 promoter, inhibiting transcription and regulating cell proliferation in spikelet glumes [191,192]. Furthermore, GLW7 and GW8 both positively control grain hull cell size, increasing grain length and yield. Small and round seed 5 (SRS5) with a single amino acid mutation (p.Arg308Leu) decreases rice grain length by inhibiting cell elongation. Although GLW7 can interact with SRS5 to regulate grain size, the exact mechanism remains unclear. Furthermore, the novel gene *OrMKK3* affects morphology and grain size, suggesting a relationship between MAPK and BR-responsive pathways with regard to grain development [193]. Moreover, LARGE2, encoding the HECT E3 UB-ligase OsUPL2, controls the rice panicle size and grain numbers, and *large2* mutants showed increased panicle size and number of seeds per panicle [194].

## 4. Seed Size Regulation by Phytohormones

**Figure 2:**
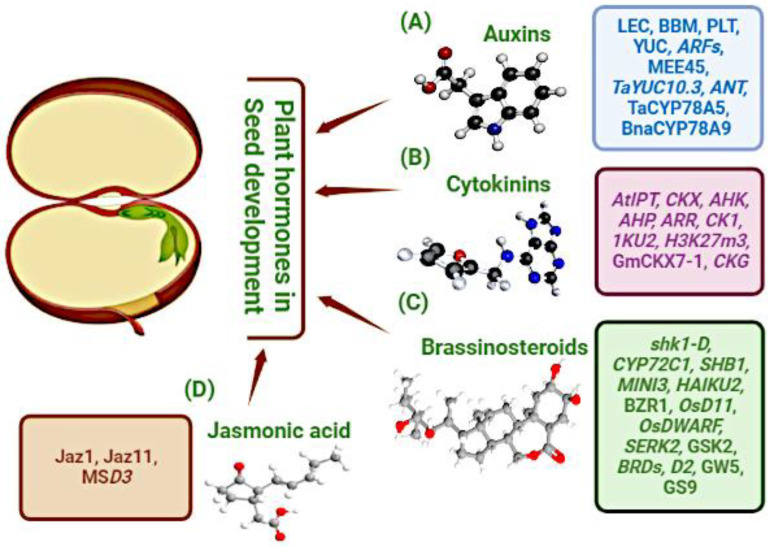
Phytohormones and their correlated molecules involved in seed size regulation: (**A**) auxins, (**B**) cytokinins, (**C**) brassinosteroids, and (**D**) jasmonic acid.

Phytohormones control a vast range of developmental and physiological functions in plants. Furthermore, phytohormone levels fluctuate at various stages in different tissues during seed development, playing a significant role in the processes involved [195]. Multi-hormonal regulation, mediated via auxins, cytokinins, brassinolides, and jasmonic acid, plays an important role in endosperm propagation and embryo growth. Phytohormones and related molecules are schematized in Figure 2.

### 4.1. Auxins

Auxin, a plant hormone, has been linked to a wide range of characteristics of plant growth, including seed production [196]. The key auxin in all plants is indole-3-acetic acid (IAA). It is biosynthesized from the tryptophan (Trp) amino acid in a two-step mechanism which is highly conserved throughout plants [197,198]. First, the amino group is removed from Trp in the first phase, which is catalyzed by transaminases from the TRYPTOPHAN AMINOTRANSFERASE OF the ARABIDOPSIS (TAA/TAR) family, resulting in indole-3-pyruvate (IPA). YUCCA is known to upregulate the auxin biosynthesis pathway and the indole-3-pyruvic acid (IPA) pathway. Previously, 11 members of YUC genes have been reported in Arabidopsis and are involved in the development of the basal body during embryogenesis [199]. Throughout embryogenesis, YUCI and YUC4 are expressed in a variety of cell types, and their expression coincides with that of YUC10 and YUC11 in developing seeds [199]. LEAFY COTYLEDON (LEC), BABY BOOM (BBM), and PLETHORA (PLT) transcription factors are known to bind or control auxin-associated genes under normal conditions, which stimulate somatic embryo development. In addition, ectopic expression of LEC2 promotes YUC2 and YUC4 expression early in the seedling somatic embryo development [200], whereas LEC1 ectopic expression promotes YUC gene expression during 2,4-D-induced somatic embryogenesis from immature zygotic embryos (YUC1, YUC4, and YUC10) and seedlings (YUC10) [201,202]. Auxin response factors (ARFs) control auxin-responsive genes’ expressions in plants [203]. ARFs are B3-like transcription factors that directly bind to the auxin-responsive element (AuxRE) and activate or repress auxin-responsive genes in plants. In Arabidopsis, twenty-three ARF proteins, including ARF2 and ARF8, have been suggested as funneling auxin signals, to control seed size by reducing cell division [204,205]. In Arabidopsis, twenty-three ARF proteins, including ARF2 and ARF8, have been suggested as funneling auxin signals, to control seed size via reducing cell division [204,205]. ARF3/ETTIN mutations cause significant polarity abnormalities in the gynoecium, including apical tissue over-proliferation and ovary development [206]. ARF6, ARF7, and ARF8 promote cell growth, and hypocotyl elongation [207]. NtARF8 is involved in NtTTG2-regulated seed development in tobacco (*Nicotiana tabacum* L.) and supports seed quantity in collaboration with NtARF17 and NtARF19 [55]. Another study identified a B3 transcriptional factor, Arabidopsis maternal effect embryo arrest 45 (MEE45), a downstream regulator of auxin biosynthesis in the ovule, which controls seed size maternally and regulates cell proliferation via the transcriptional activation of aintegumenta (ANT). In this case, the largest seed size was observed for MEE45 expression, whereas the mee45 mutant expression resulted in reduced seed size [208]. Auxin also regulates the growth of siliques, which is noteworthy. Overexpression of BnaA9.CYP78A9, a member of the P450 monooxygenase gene family, stimulates the elongation of *Brassica napus* siliques by causing a significant increase in auxin [209]. However, most recently the modified expression of TaCYP78A5 has been shown to increase grain weight and yield per wheat plant by accumulating auxin [210]. Hence, the aforementioned studies based on transgenic and mutant studies imply that the auxin biosynthesis pathway, signaling, and transporter genes are essential in regulating the auxin levels during seed development, controlling embryogenesis, endosperm development, and seed coat development.

### 4.2. Cytokinins

Cytokinins (CKs) are phytohormones involved in the modulation of cytokinesis-related enzyme activity and CK-mediated signaling. Additionally, CKs are important for initial endosperm cellularization [211]. The discovery of CK-catabolizing genes, in addition to their distinctive genetic and biochemical properties, has introduced the role of CKX enzymes in CK regulation [212], as well as multi-gene families synthesizing CKX enzymes. Various studies have aimed to determine the role of CKX family members (GFMs) in improving grain yield in many crops, including barley [213,214], rice [159,215], and wheat [216,217]. A transcription factor basic helix–loop–helix, cytokinin growth regulators (CKG) promote CK-mediated regulation of cell expansion and cell cycle development in Arabidopsis. CKG expression has been noted to enhance the size of cotyledons during the reproductive stage, whereas expression of ckg mutants has been noted to mediate the opposite effect [218]. Another study elucidated that CK activity is primarily regulated by the transcription factor receptors ARR1 and AHK3. Similarly, increased levels of CK in the external layer of reproductive tissues and the placenta lead to larger siliques and increased ovule formation, thereby increasing the number of seeds and resulting in higher seed yield [219]. CKs have also been observed as methylthiolated derivatives (MET); however, details of the function of this group of compounds are scarce [220]. The level of CKs during seed development is controlled by balancing the activities of isopentenyl transferase (IPT) and CK oxidase/dehydrogenase (CKX), which play an important role in the biosynthesis and degradation of CKs, respectively. AtIPT4 and AtIPT8 are expressed at higher levels during seed development in the chalazal endosperm area, during morphogenesis [221,222]. Furthermore, Song et al., 2015 suggested that silique and growing seeds have the ability to synthesize CK [223]. The endosperm-specific expression of IPT increases the levels of CK to grow seeds and seed mass without causing any morphological aberrations in transgenic tobacco plants [224]. Numerous studies have reported that higher CK levels occur during development, upon external application, and that ectopic exposure of IPT is linked with increased CK degradation resulting from increased CKX gene expression [225,226]. Furthermore, the expression of the CKX and IPT GFMs suggests a positive correlation between the two in wheat [227] and *Brassica napus* [223].

CKs have a role in transcription control, and different genes in Arabidopsis influence the activation of its receptor histidine protein kinases (AHKs). AHKs activate AHPs, which are histidine phosphotransferase proteins, in response to CKs, and AHPs stimulate Arabidopsis response regulators (ARRs), thereby inducing the expression of all genes. Triple mutants, ahk-1,2,3 and ahp-1,2,3, have been observed to enlarge the embryo and seeds [221]; moreover, the same study concluded that cytokinin independent 1 (CKI1) expression plays an important function in CK signaling. CKI1 contains a histidine kinase-deficient CK perception domain of AHK. The cki mutants’ expression results in fewer seeds that are larger in size, thereby suggesting CKI1 as playing a function in determining seed number and size during seed development [228]. Moreover, Li et al., 2013, elucidated that the expression of cytokinin oxidase 2 (CKX2), which encodes a protein that induces CK degradation, controls endosperm development [89] and up-regulates the CKX2 expression, is controlled by IKU2 and H3K27m3 [86]. These experimental findings explain that CK expression controls seed development phases through members of the CK signaling pathway and other epigenetic approaches [229]. Another study identified 17 GmCKX GFMs in soybean, and natural alterations were probed among cultivars with different yields. A total of 5 out of 17 CKX genes were noted to be responsible for the regulation of CK content during the grain-filling stage, as indicated by the results of introducing single nucleotide polymorphisms (SNPs) in them. GmCKX7-1 was discovered to contain a non-synonymous mutation (H105Q) on histidine 105, one of the amino acid residues in the active site, during the critical grain filling period, to preserve structural reliability of the enzyme, consequently enhancing seed yield [66].

### 4.3. Brassinosteroids

Brassinosteroids (BRs) are a recently documented class of phytohormones that play significant roles in plant growth and development, such as cell elongation and division [230,231,232]. BRs mainly take part in regulating the yield determination of agriculture traits, including seed size regulation [233]. In Arabidopsis and rice, molecular analysis of BR-defective mutants permitted the identification of very important genes involved in the BR-mediated regulation of seed development. The DEP1 promoter has been revealed to increase rice grain size and yield by optimizing the expression of BR-like genes [234,235]. Three steps are involved in the synthesis of BR from campesterol: (1) formation of campestanol from campesterol; (2) two concurrent routes from campestanol to castasterone (CS); and (3) B-ring lactonization of CS to 24-epibrassinolide (BL). OsDWARF (OsBR6ox) is a crucial gene in the biosynthesis of BR. The OsDWARF product catalyzes a late stage in bioactive BR formation via combining the early and late C-6 oxidation pathways and encodes BR-6-oxidase, which can convert 6-deoxotyphasterol (TY) and 6-deoxoCS to TY and CS, respectively [236,237]. The OsDWARF loss of function produces a decrease in TY and CS, resulting in the BR-deficient phenotype. Further, dwarf mutant shrink1-D (shk1-D) exhibited a lower level of BRs, which caused reduced seed length in Arabidopsis. In the case of shk1-D mutants, CYP72C1 overexpression and hydroxylation of BRs resulted in low endogenous BR levels and decreased seed size [235]. Furthermore, putative brassinolide receptor brassinosteroid in-sensitive1, a defective mutant, also caused lower seed production [226]. Presumably, this receptor activated the expression of genes encoding BRs that act as positive regulators of seed size (HAI-KU2. MINI3 and SHB1) and inhibited that of genes encoding negative regulators of seed size (ARF2 and AP2) through the TF brassinazole resistant 1 (BZR1) [238]. Higher expression of BR synthesis-related gene OsD11, or that of BR signaling factor OsBZR1 induced an increased sugar-accumulation rate in developing seeds and increased grain yield in rice. By contrast, the knockdown of these genes cleared the imperfect pollen, seed size, and weight reduction compared with the control [239]. In the BR signaling pathway, cytochrome P450 mutant key enzyme D2 (D11) [234,240], BR-deficient dwarf 1 (BRD1) [236], DWARF 2 (D2) [241], and other genes related to BR biosynthesis, including BRD2 [242], all have very close phenotypes, such as decreased plant height, shoot length, spikelet length, and grain size. Furthermore, GW5 also interacts with and suppresses the activity of glycogen synthase kinase 2 (GSK2), a crucial kinase involved in BR signaling. The BZR1 and DLT transcription factors, downstream of GSK2, enter the nucleus in an un-phosphorylated state to modulate BR signaling-responsive genes, thereby affecting grain size in rice [120,226]. Moreover, GW5 and GW5-related proteins physically bind to the important components of the BR signaling pathway, GSK2, and BIN2, resulting in the accumulation of un-phosphorylated DLT and OsBZR1 [243]. Additionally, GSK2 binds to and phosphorylates members of the OVATE family protein 8 (OFP8) [244]. Furthermore, OFP8, OFP14, and the transcriptional activator GS9 interact with one another. OFP14 inhibits GS9 transcriptional activity, and GS9 overexpression causes circular grain shapes with BR-defective phenotypes. As a result, GS9 appears to negatively regulate the BR signaling [245]. In yeast two-hybrid screens, OFP3 binds to both GSK2 and DLT and negatively regulates the BR response, in addition to OFP8 and OFP14 [246]. Plants with the dss1 mutation were dwarf and showed erect leaves and smaller seeds. Similar results were observed in BR-deficient mutants [247]. In addition, SERK2 has been reported as a BR signaling element for many agronomic traits, especially yield and stress-related traits. Moreover, SERK2 knockdown mutants showed enhanced rice grain size [248].

### 4.4. Jasmonic Acid

Jasmonic acid (JA), a phytohormone produced by free α--linolenic acid, is similar to animal prostaglandins [249,250] and serves a variety of functions in plant response to biotic and abiotic stressors as well as development [226,251,252]. JA influences pollen formation or pollen shedding [253], embryo and seed development [254,255], spikelet formation [256], and maize sex determination [257,258,259], during inflorescence development. Linolenic acid is the substrate of JA biosynthesis, which is then metabolized to produce the bioactive isoleucine conjugate, JA-Ile [250]. The interaction of JA-Ile with the COI1 receptor (CORONATINE INSENSITIVE1) activates JA signaling, resulting in the proteasomal degradation of JAZ (JASMONATE ZIM-DOMAIN) transcriptional repressor proteins [260,261]. JAZ repressor degradation releases a number of transcription factors, especially MYCs, which interact with MED25/PFT1 to negatively regulate seed size [262]. The eg1 (extra glume 1) JA biosynthetic mutant displayed aberrant spikelet formation. The gene EG1 encodes a plastid-targeted class I lipase, which is required for the production of JA precursors. Furthermore, eg2-1D showed disrupted floral identity as well as impairment in floral meristem determination [256]. Map-based cloning showed that EG2 encodes for OsJAZ1, a JAZ repressor. Another comprehensive investigation revealed that the spikelet defects in eg2-1D are caused by the inhibited function of the OsMYC2-controlled E-class gene, OsMADS1. In addition, OsJAZ11 regulates the width and weight of rice seeds. When compared to wild-type, transgenic rice lines overexpressing OsJAZ11 showed up to a 14% increase in seed width and a 30% increase in seed weight. The constitutive expression of OsJAZ11 had a significant impact on spikelet development, resulting in additional glume-like structures, an open hull, and an abnormal number of floral organs. Also, transgenic lines accrued increased JA levels in spikelets and developing seeds. Overexpression lines exhibited altered expression of JA signaling and MADS-box genes when compared to WT [263]. According to yeast two-hybrid and pull-down experiments, OsJAZ11 interacts with Os-MADS29 and OsMADS68. Surprisingly, in overexpression lines, the expression of OsGW7, an important negative regulator of grain size, was dramatically decreased. Furthermore, multi-seeded 3 (msd3) sorghum mutants that can quadruple grain number per panicle by expanding panicle size and modifying floral development such that all spikelets are viable and set grain, in turn, decreased the JA levels [263].

## 5. Possible Strategies Used to Maintain Seed Size

Increasing grain yield is always an important goal in plant breeding programs, especially in the case of domesticated crops. Additionally, increasing seed size without affecting seed number via conventional plant breeding programs has allowed only limited progress because of the trade-off between these two yield components [264]. Many researchers have indicated that modern technology, including transgenic technology, genome editing, marker-assisted selection (MAS), and genomic selection (GS), has major challenges regarding the disruption of grain yield in different crops. The use of molecular markers for plant breeding selection has resulted in major advancements in efficiency and the successive announcement of many new varieties in the last 30 years [265,266]. MAS is often more effectual than traditional methods, resulting in improved accuracy, charge, or time savings, or the ability to screen for diseases that would otherwise be impossible to detect using standard phenotyping methods [267]. One of the major benefits of markers is that homozygosity can quickly be detected. In the private sector, molecular breeding programs have been widely adopted, with reports indicating increasing rates of genetic gain [268,269]. Numerous reports of marker-assisted variety improvement have emerged as a result of the widespread use of MAS in major crop breeding programs [270]. Backcrossing with DNA markers significantly improves selection efficiency. In essence, marker-assisted backcrossing (MABC) allows for very effective detection of the most important genes of interest or QTLs while preserving the recipient variety’s original important features, allowing the original variety to be “upgraded” [265]. Whole-genome-based molecular breeding methodologies have been developed as a result of advances in rice genomics. Genomic selection (GS) is a new approach that has recently evolved [271]. Genomic selection, like MAS, is based on making predictions on a genomic scale from many DNA markers rather than focusing on individual genes or QTL [272,273]. Over the last ten years, pilot research in rice, wheat, and maize has yielded promising results in reducing breeding cycles and speeding up variety creation. Genomic selection provides a lot of potential for accurate selection of complicated variables such as yield and shortening the breeding cycle to improve genetic gain [274]. Recently, it has been reported that the *GW6* gene is better for increasing grain length and width than Baodali, which was transferred into the recurrent parent 9311 *indica* cultivar and Zhonghua 11 cultivar *japonica* via MAS. Near isogenic lines (NILs) of these two cultivars displayed improved grain weight and grain production [275]. In addition, most studies have suggested that the pyramiding of grain size is a convenient method for QTLs to boost grain production in different plant species. For example, a study found that the pyramid of grain size and weight was better through QTLs and the involvement of the NILs NIL-qGL3, NIL-*qGW2a*, and NIL-*qGS5*. Similarly, the implementation of NILs in the genetic study of Zhenshan 97 using MAS and conventional back-crossing has been reported. The combination of three major QTLs for grain size showed improved effects on grain weight without any type of interaction [276]. A similar study reported that NIL*GS3*/*qgl3* has been established via crossing NIL-*GS3* with NIL*qgl3* by the MAS approach. The *GS3* and *qGL3* combined effects on grain length were compared among NILs. Furthermore, primary panicle transcription analysis in NILs showed that the gene expression regulated by *GS3* and *qGL3* did not overlap [277].

Fine-mapping and cloning for crop production, particularly grain weight, have made significant improvements in the last two decades. To date, 20 QTLs have been cloned for grain size and weight, including GL7/GW7 and GS9, which have opposite allelic directions of additive effects on grain length and weight, controlling grain size but having little effect on grain weight [192,278,279]. GSA1 and GW6a have similar results on grain length and weight with similar directions, implying that they significantly impact grain weight [13,280]. The remaining 16 QTLs have an impact on both grain size and grain weight. GW2 [281], TGW2 [282], GS5 [283], qSW5/GW5 [120,121], GW6 [284], and GW8 [191], are six that largely control grain width and weight, while the other ten QTLs containing GS2/GL2 [177,178], OsLG3 [285], qLGY3/OsLG3b [158,286], GS3 [153], GL3.1/qGL322 [287], TGW3/GL3.3 [288,289], GL4 [290], TGW6 [291], GL6 [292] and GLW7 [49] largely control grain length and weight. All these QTLs characterizations have greatly aided our understanding of the genetic regulation of rice grain size and weight, but further research is required to further improve our understanding of the regulatory mechanism for these important agronomic traits [111].

Numerous studies have reported that alterations in the regulatory genes of quantitative traits, such as oil accumulation in the seed, showed a significantly increasing trend in intrinsic yields [293,294,295]. Selective genes that have played a vital role in seed development at different developmental phases require expression data of different developmental stage-specific tissues [296]. Furthermore, in polyploid species, the selection of target genes involved in seed size engineering may require specific tissue- and different stage-specific expression outlines of separate GFMs [297]. New technologies in functional genomics have been introduced for the development of seeds, and genetic engineering approaches provide greater scope for engineering to control seed size. Furthermore, increasing evidence shows that a thorough molecular knowledge of seed formation could yield opportunities for controlling seed size in plants [14,234,298]. For example, in a field study, the downregulation of *BnDA1* in *B. napus,* the *AtDA1* ortholog (a harmful regulator of seed formation)*,* improved seed weight and seed yield by 21% and 13%, respectively [14]. In Arabidopsis, the two genes, *AtSHB1* and *AtKLUH,* involved in the seed development were introduced in *Brassica juncea* and improved seed weight by 40% [299]. *AtSHB1* overexpression in *B. napus* increased seed size 1.6-fold [300]. The loss of *ARF18* function mutant in *B. napus* showed an approximately 8% increase in seed size compared to WT plants [301].

Grain size and yield increase in rice crops through the response to auxins by *big grain 1* (*BG1*), encoding a membrane-connected protein (increased by about 15.2% in length and 17.0% in width, respectively). Furthermore, RNAi suppression of *BG1* led to a decrease in rice grain size and yield relative to WT plants [301]. Downregulation of *big seed 1* (*BS1*) and *BS2* genes in soybean and *Medicago* resulted in significant enhancement of seed size [302]. Engineering techniques that are better for achieving larger seed size involve the overexpression of important regulators for the development of seeds in Arabidopsis [14,303] and other respective species [298] or the silencing of orthologs that are harmful regulators of seed growth [302]. Genetic material regulating biosynthesis, metabolism, and signaling is useful for gene manipulation, altering hormonal regulation, and producing larger seeds. For example, *BZR1*, a BR-responsive TF expressing most of the positive regulators, *SHB1* and *IKU2*, while *AP2* and *ARF2* are harmful regulators [238], may be modified in a spatiotemporal manner. Epigenomes are used as a genome editing technique in locus-specific ways and via methylation or de-methylation. Furthermore, several target genes that control the organized growth of maternal and zygotic tissues could be manipulated to increase seed size and crop yield. For example, KLUH and SHB1 regulate the development of seed coat and endosperm, and embryo formation, in a specific spatiotemporal manner, respectively. Moreover, control of cell cycle core gene expression is an alternative approach to increase seed size and yield. For example, target-specific up-regulation of the core cell cycle component CYCD7;1 in the endosperm helps overcome incomplete seed termination interlinked with ectopic expression [104]. Furthermore, genetic manipulation of genes that govern seed traits may reduce the negative relationship between the number and seed size. In Arabidopsis, FATA2, BZR1, LecRK-VIII.2, and CKX2 expression controls seed number, and expression of many other genes increases seed size and yield.

**Figure 3:**
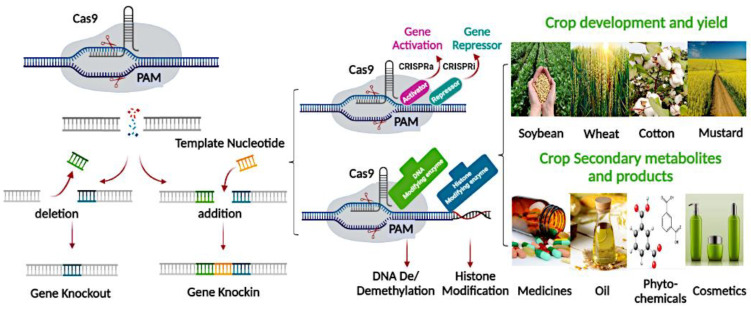
Application diversity of the CRISPR/Cas system for functional genomic research and crop improvement. CRISPR may directly generate gene knockout (silencing) via deletion or addition with a number of bases and repair through NHEJ depending on the DNA double-strand break mechanism. Alternatively, genome editing may replace an undesirable gene and/or overexpress (knock-in) a single gene when homolog-directed repair occurs with a DNA donor. In addition, CRISPR/Cas may also be employed for base editing, and epigenome editing by deactivating the Cas9 enzyme while using transcription effectors or other enzymes coupled with the dCas9.

Genome editing technology, especially the CRISPR/Cas9 editing system, has widely been used in many plant species to knockout an individual gene, the key objective of its editing and repair mechanisms (Figure 3). The widely employed CRISPR/Cas9 and various CRISPR/Cas systems often generate a double-strand break (DSB) by cutting the double-stranded DNA. The double-strand break will often be repaired using non-homologous end joining (NHEJ). Because one or more nucleotide deletions or insertions may inhibit targeted gene expression due to frame shifting in the coding region, genome editing typically results in either gene knockout or silencing. CRISPR/Cas-induced gene silencing has many advantages over T-DNA mutagenesis. The main advantage of CRISPR/Cas-based editing is its specificity; it may be used to target a particular gene without having any negative effects. Agrobacterium-mediated gene transformation has largely been used to overexpress a specific gene in a plant cell via T-DNA implantation, which includes the foreign DNA sequences. Even so, the haphazard insertion of a specified gene into a genome always has numerous adverse effects, such as the silencing of other useful genes. The CRISPR/Cas system may cleave the DNA sequence at a specified location, and homolog-directed repair (HDR) will then insert a targeted gene sequence into such a cleavage position. As a result, CRISPR/Cas may be used to accurately insert a gene of interest into a particular site inside a genome, avoiding interfering with neighboring genes or preventing position effects. Base editing (BE) is also a new technology that can specifically and proficiently implant point mutations at target positions without the use of DSB formation and donor DNA templates [304]. The cytosine base editor (CBE) and adenine base editor (ABE) are common examples of base editing technology, which has been applied in various plant species to interrogate gene function for crop trait improvement [305,306].

In gene replacement for gene mutations, as in the assembly of HDR, any sequence may be inserted and replace the existing genomic sequence surrounding the DSB locations. Thus, if the nucleotide sequences have mutations, including point mutations or other undesired sequences, we may replace them with suitable nucleotide sequences that have homologous arms with the existing sequences. It was recently reported that transcript-template HDR was employed to completely replace the rice aceto-lactate synthase gene (ALS) by transport of such a DNA-free ribonucleoprotein complex [307]. Prime editing (PE) is a new DSB-independent precise genome editing technology that can introduce any base conversion, small indels, or a combination thereof, at target positions [304]. The prime editing approach was initially established in the animal system but rapidly utilized in plants to edit a precise gene. Prime editing is now being used to effectively replace genes in numerous key plant species, such as Arabidopsis, tobacco [308], rice [309,310,311,312], wheat [313], maize [314], tomato [315], and potato [316].

A CRISPR/dCas9 interference (CRISPRi) system was developed based on the application of the CRISPR/dCas9 system to disrupt transcriptional activities and to be used as a gene knockdown strategy for controlling gene expression [317,318]. CRISPRi could be used to inhibit the expression of genes by restricting transcription start and/or elongation depending on where the CRISPR/dCas system interacts. When the Cas system connects to the upstream area, it will impede TF and RNA polymerase (RNAP) binding and restrict transcription initiation. However, when it attaches to the coding region, it will prevent RNAP interaction and restrict transcription elongation [318]. Regulating a gene’s methylation and demethylation can be an effective strategy to influence the expression and, ultimately, trait regulation. As a result, since the CRISPR/Cas system was successfully permitted as an effective genome editing system, research efforts have focused on tweaking it to alter DNA methylation.

CRISPR/Cas9 has great potential for specifically targeting desired genes [319]; thus, it could be used to develop crops with the desired seed size by knocking out specific genes without altering other traits [320]. However, in some cases, seed size may be linked with other important traits. For example, CRISPR-Cas9 genome editing of the TaIPK1 gene in wheat enhanced nutritional value and increased seed size simultaneously [321]. Recently, using CRISPR/Cas9-based genome editing, it was found that the MADS-box TF genes MADS78 and MADS79 are very important for regulating endosperm cellularization and early seed formation in rice [76]. A single MADS78 or MADS79 knockout mutant displayed early endosperm cellularization, while double mutants hindered seed development and produced no viable seeds [76]. Most recently, genome editing of cis-regulatory elements (CREs) has been found to result in both gain- and loss-of-function alleles, which is proving very useful for broadening the phenotypic range of traits related to yield and architecture [322,323].

## 6. Conclusions and Future Recommendations

Seed size and seed weight regulated by seed development are critical determinants of crop yields. Seed size can be determined through the development of zygotic embryos and endosperm as well as maternal tissues. Many signaling pathways that may determine seed size by the development of endosperm and maternal tissues, such as the IKU pathway, MAPK signaling, G-protein signaling, ubiquitin–protease signaling, etc., induce significant effects on entire features of the growth and development of plants, as well as regulating seed size. Transcription factors are responsible for enhancing cell growth in the maternal ovule and affect seed size. Therefore, there is a need to increase seed size with no effect on seed number through convention breeding programs to improve crop yield.

Many researchers have indicated that modern types of molecular approaches i.e., MAS, GS, and CRISPR/Cas system-based editing and transgenic technology have limitations to their disruption of seed yield hurdles in different plant species. In this review, we summarized many factors regulating seed sizes, such as genetic factors, signaling pathways, and transcriptional factor regulators in Arabidopsis and other crops, followed by engineering seed size by using recently evolved novel transgenic and breeding techniques, and ending with a brief discussion on recent studies, and those conducted over the last decade, which aimed to comprehend the genetic and molecular aspects controlling seed size. However, more research studies are required to understand the seed development pathways via molecular and genetic factors. Moreover, there is a need to introduce more strategies for genetic modifications to improve grain size and yield.

## Figures and Tables

**Table 1:** List of reported genes that control seed size and yield in different plants.

Species	Genes or Mutants	Function	References
Arabidopsis	*IKU1*, *IKU2*, and *MINI3*	Early cellularization of endosperm produces smaller seeds	[19,20]
Arabidopsis	TFL1	Timely endosperm cellularization enhances seed size	[21]
Arabidopsis	*SHB1*	Up-regulates the functions of *IKU2* and *MINI 3* and postpones endosperm cellularization, producing larger seeds	[22,23]
Arabidopsis	TTG2	Identifies integument cell elongation and endosperm growth as the primary regulators of seed size	[24]
Arabidopsis	*TOP1α* and *UPF1*	The molecular interplay among these three genes explains their biparental gametophytic effect on seed size regulation	[25]
Arabidopsis	LuLEA1	Negatively regulates seed size and yield	[26]
Arabidopsis	AN3-YDA	Disrupts normal sucrose and glucose contents and alters seed size regulation in *an3* or *yda* mutants	[27]
Arabidopsis	EIN_3_-YDA	Both are integral to a sugar-mediated metabolism cascade that regulates seed mass by maternally controlling embryo size	[28]
Arabidopsis	DME	Enhances DNA *de-*methylation activity and endosperm chromatin composition	[29]
Arabidopsis	CYCB1;4	Overexpressed in transgenic plants to show increased seed size in response to faster cell cycle progression to increase grain size and yield	[30]
Arabidopsis	FIS2, FIS3, FIE, MEA, and MSI1	Control endosperm development	[31]
Arabidopsis	*AGL62*	Regulates endosperm development by the *FIX*	[32]
Arabidopsis	*PHE1*	Controls seed abortion by *mea* mutant	[33]
Arabidopsis	CDKs and CYC	Control cell cycle development	[34]
Arabidopsis	*RBR*	Decreases the cell proliferation in the integuments and stops further development of seed coat	[35]
Arabidopsis	AtENO2	Decreases the size of seed and mass with the declined concentration of cytokinin	[36]
Arabidopsis	*MET1* and *DDM1*	DNA methylation of maternal and paternal genome regulates seed size	[37]
Arabidopsis	*FIS2*, *FWA*, and *MPC*	Regulate parental imprinting by DNA methylation	[38,39,40]
Arabidopsis	*MEA*	It plays the role as a maternal buffer of paternal effects on seed development	[41]
Arabidopsis	*PRC2*	Controls turgor pressure during seed development	[42]
Arabidopsis	AtSOB3	Produces heavy and larger seeds with long hypocotyl	[43]
Arabidopsis	p4-siRNAs	Expressions are observed in maternal gametophytes and continue until endosperm development	[44]
Arabidopsis	CEPR1	Promotes reproductive parts including seed size and yield	[45]
Arabidopsis	*CYCD3:1* and *CYCD7:1*	Promote early endosperm and embryo development	[46,47]
Arabidopsis	AtbZIP75	Regulates seed development	[36]
Rice	*OsEMF2a*	Delayed cellularization and autonomic endosperm development	[48]
Rice	GLW7	Controls grain size and yield	[49]
Rice	OsGW2	Regulates grain size and reduces rice yield	[50]
Rice	*GS3*	Regulates grain length	[51]
Rice	*OML4*	Negatively regulates seed size by terminating cell expansion	[52]
Rice	*qGL7*	Could display a single mendelian characteristic, suggested as producing diverse seed shapes	[53]
Rice	qGL11	Regulates rice grain weight and length	[18]
Rice	*MIS2*	Controls grain size by coordinately regulating epidermal cell size and cell number	[54]
Rice	MADS78 and MADS79	Regulate endosperm cellularization and early seed formation in rice	[55]
Maize	miRNAs	Control embryogenesis via transcriptional regulation	[56]
Maize	*GSI*, *qZmGW2-CHR4*, *zMgs3*, and *qZmGW2-CHR5*	Control kernel size and grain production	[57,58,59]
Maize	*ZmBZR_1_*	It attaches to the promoter region of *GRACE* and *KRP6* to control their expression and regulate seed size	[60]
Wheat	*TaGW2*	Controls grain weight	[61]
Cotton	*MicroRNA160*	Regulates smaller seed size	[62]
Bean	SL9.1 ^GA^	Controls seed length	[63]
Rapeseed	*BnaEOD3*	Negatively regulates the seed growth and development	[64]
Soybean	*GmSWEET10a* and *GmSWEET10b*	Change seed size, oil quantity, and protein contents	[15]
Soybean	qSL-13-3_zy_, -13-4_zy_, and qSW-13-4_zy_	Control seed size and yield	[65]
Soybean	GmKIX8-1	Increases cell proliferation and size of aerial plant organs such as leaves and seeds with high expression of *CYCLIN D3;1-10*	[66]
Peanut	SNE and SAP	Regulate seed and fruit size	[67]
